# AQFormer: severity-aware transformer with aphasia-specific CAM for spoken keyword classification in aphasic speech

**DOI:** 10.3389/frai.2026.1786757

**Published:** 2026-05-08

**Authors:** Gowri Prasood Usha, John Sahaya Rani Alex

**Affiliations:** School of Electronics Engineering, Vellore Institute of Technology, Chennai, Tamil Nadu, India

**Keywords:** aphasia, AphasiaBank, class activation mapping, explainable AI, film, keyword classification, severity-aware modeling, transformer

## Abstract

Language-driven speech output in individuals with aphasia shows considerable variability, including phonological errors and pauses during word searches. This makes it difficult to use traditional keyword classification systems and further reduces trust in deep neural models, complicating their application in clinical settings. This paper introduces AQFormer, a severity-aware transformer architecture designed to classify spoken keywords in aphasic speech, and A-CAM, a dual-stream attribute framework aimed at assisting individuals with aphasic impairments. AQFormer generates acoustic representations that are severity-adaptive by integrating patient-level Aphasia Quotient (AQ) scores through Feature-wise Linear Modulation (FiLM) and A-CAM. A-CAM consists of two main components: (i) a branch that influences WavLM convolutional features, a prediction-focused one, and (ii) a multimodal aphasia filter that captures pauses, phoneme variations, and interruptions at word boundaries, an impairment-focused branch. We introduce an adaptive perturbation and dual-filtering gradient scheme that enforces non-negative, mask-consistent attributions over time-frequency regions. Experiments utilizing a subset of AphasiaBank keywords (93 speakers, 960 recordings; training set expanded to 5,138) with rigorous speaker-disjoint evaluation indicate that AQFormer achieves approximately 96.61% accuracy (F1 = 96.8%) on previously unseen speakers. A-CAM consistently outperforms several Grad-CAM variants when deletion/insertion AUPC and ADCC metrics are employed. This results in stable, sparse explanations that reflect how aphasia is usually caused: Discriminates correct from incorrect productions with Cohen’s *d* = 2.05 (a massive effect size) and spatial localization of error regions with Intersection over Union (IoU) of 0.461 against phoneme boundaries. Montreal Forced Aligner meets the quantitative validation criteria for the aphasia filter. The impairment-focused A-CAM maps achieve an IoU of 0.712 against detected error regions, with a severity correlation that doubles from rho = −0.374 (base) to rho = −0.754 (filter-gated). By tightly coupling severity-aware modelling with aphasia-informed attributes, the proposed framework advances explainable learning systems for aphasia-affected speech without needing clinician-labelled training targets.

## 1 Introduction

Aphasia, a language disorder that severely affects language processing, poses a significant barrier to spoken communication (Brady et al., 2016). Language impairment involves difficulties with verbal and written expression, comprehension, or both. Most cases of aphasia possess a combination of these issues, affecting various language functions. The most prevalent aphasia varieties are Broca, Wernicke, conduction, transcortical, Anomia and alexia, with or without agraphia. The primary cause is usually stroke, particularly ischemic stroke, but it can also arise from traumatic brain injury, tumors, or neurodegenerative diseases. Symptoms range from mild word-finding difficulties to severe language impairments. The Boston Diagnostic Aphasia Examination (BDAE) (Kaplan et al., 2016) and the Western Aphasia Battery (WAB) (Kertesz, 2022) are the standardized assessments that include neuroimaging. Treatment focuses on addressing the underlying cause and often involves speech therapy and support for associated health issues, highlighting the importance of a collaborative healthcare team in managing aphasia (Le et al., 2024).

Speech-language pathologists need robust tools for evaluating and classifying spoken keywords in aphasic speech in order to support personalized therapy and progress tracking, enhance communication aids, and improve interaction with technology (Kiran and Thompson, 2019). Individuals with this aphasia often know what they want to say but have difficulty retrieving the appropriate words, frequently experiencing a ‘tip of the tongue’ phenomenon. Their language output may contain errors such as semantically (semantic paraphasia) or phonemically (phonemic paraphasia) related words or even a non-word (neologism), and the frequency of these errors can also vary significantly both within a single individual and among individuals with aphasia (Barbera et al., 2021). It is important to distinguish language errors (e.g., paraphasia arising from impaired lexical-semantic or phonological processing) and speech errors (e.g., articulatory distortions arising from impaired motor planning, as in apraxia of speech). Aphasia primarily involves the former, although both may co-occur. This marked variability in speech patterns makes the use of typical speech recognition systems particularly challenging in aphasia (Barbera et al., 2021; Wade et al., 2001).

Traditional aphasia diagnosis and classification approaches have primarily depended on statistical models and rule-based methods (Akbarzadeh-T and Moshtagh-Khorasani, 2007), which use manually designed features such as phoneme patterns and temporal cues (Danly and Shapiro, 1982; Ash et al., 2010). However, the highly variable and unpredictable nature of aphasic speech limits the effectiveness of these methods, particularly in handling distortions, deletions, substitutions, and timing abnormalities associated with non-canonical speech production. Although such approaches can still perform well under controlled conditions, their robustness in real-world aphasic speech analysis remains limited (Konstantinopoulou et al., 2019).

Traditional Spoken Keyword spotting (KWS) focuses on detecting the presence of a specific keyword in an audio stream or spontaneous speech. It typically answers the question: “Is this keyword present in the present audio or not?” (Shan et al., 2018; Lopez-Espejo et al., 2021; Sun et al., 2024). This task is challenging in aphasia speech due to phonological errors, semantic errors, unstable prosody, frequent pauses, and grammatical disruptions, all of which hinder robust generalization. Prior studies have explored hybrid HMM/MLP and bidirectional GRU-based systems for aphasia-oriented KWS and utterance verification (Abad et al., 2013; Barbera et al., 2020), but these approaches remain limited in handling severe variability and do not provide clinically meaningful interpretability. In contrast to binary keyword spotting, the task addressed in this work, spoken keyword classification, requires identifying which target word a speaker with aphasia attempted to produce from a closed set of naming test items. This multi-class formulation is directly aligned with clinical naming assessments such as the BNT, where the goal is not merely to detect speech activity but to evaluate lexical retrieval accuracy across varying severity levels.

Recent advancements in Artificial Intelligence (AI) indicate that pre-trained language models can aid in aphasia rehabilitation at the linguistic level. They offer ongoing, understandable metrics for tracking treatment progress (e.g., PLM-derived surprisal) and facilitate automated evaluations of sentence accuracy and error types through prompt-based approaches (Cong and Lee, 2025). To accelerate the conversational agent development for aphasia therapy, simulation methods have been proposed (Imaezue and Marampelly, 2025). Methods aim to generate synthetic transcript data to address data scarcity in aphasia research (Pittman et al., n.d.) and for aphasia type detection, integrating co-speech gestures with linguistic features, using multimodal approaches has shown promise (Lee et al., n.d.). Approaches operating at the acoustic keyword level, along with severity-aware classification and disorder-informed explainability, will complement these directions.

The black-box nature of deep learning makes it challenging to apply in clinical applications where safety is crucial, even though it excels at tasks such as vision, language, and audio. This motivates us to provide clear and correct evaluations by creating a severity-aware, aphasia-informed model. For aphasia-related keyword classification, explainable AI (XAI) is required to expose the evidence underlying the model’s decisions and ensure that predictions align with clinically meaningful speech patterns.

XAI is essential in applications where comprehending the reasoning behind model decisions is just as crucial as the decisions themselves. Studies recognition but XAI techniques like Local Interpretable Model-Agnostic Explanations (LIME), and SHAP Shapley Additive exPlanations (SHAP) in audio processing (Wu et al., 2023; Akman and Schuller, 2024), for sound classification and speech recognition, however, they are restricted to communication that is healthy. By incorporating XAI into audio classification for aphasia, clinicians can gain a better understanding of the model’s focal areas, which in turn helps them trust and interpret its predictions. Although existing XAI techniques can guide the development of more effective and personalized therapeutic interventions, thereby improving patient outcomes, this could be achieved through user interfaces (Holzinger et al., 2017).

Among available XAI techniques, Gradient-weighted Class Activation Mapping (Grad-CAM) is among the most popular methods for classification tasks across application domains. The current technique successfully creates a visual explanation for image or object detection. However, it often lacks the detailed precision required for the specific area (Ali et al., 2023) like impaired speech. So, developing a variant of the current model by appending or integrating new, intricate elements would be feasible, and these elements can be recognized as novel discoveries (Bharati et al., 2023). Such improved models should be similar to previous demonstrations and models, while clearly explaining how the supplementary elements relate to previously overlooked discoveries (Bharati et al., 2023).

This study focuses on isolated spoken keyword classification using items from the Boston Naming Test (BNT), a commonly used standardized confrontation naming evaluation in aphasia clinical practice. Naming therapies, objective scoring, tracking therapy sessions, lexical retrieval success, and speech-based monitoring tools for remote rehabilitation can be implemented by automatically classifying naming responses.

While traditional aphasia syndrome classification relies on patho-linguistic features grounded in the Wernicke-Lichtheim-Geschwind model, characterizing impairment along dimensions of fluency, comprehension, and repetition (Le et al., 2024), our task is fundamentally different: we aim to recognize ‘what keyword was spoken’ from the acoustic signal, not to classify ‘what type of aphasia’ the speaker has. For the classification assignment, acoustic features are the most natural modality to use. Integrating clinical severity via the Aphasia Quotient (AQ) score in FiLM conditioning enables the model to adjust its feature representations by connecting the acoustic and clinical domains according to impairment severity. We note that our framework currently models acoustic-phonological production errors (phonemic distortions, pauses, and boundary disruptions) but does not explicitly address semantic paraphasia, neologisms, perseveration, or apraxia of speech features.

In this work, we address spoken keyword classification in aphasic speech with a unified, severity-aware and explanation-driven framework. (i) Personalized patient modelling conditioned on AQ, along with a transformer backbone (ii) highlighted decision evidence and impairment patterns using dual-stream Aphasia-specific CAM (A-CAMs), and (iii) incorporates pause, phoneme errors and word boundary errors into the attribution process from metadata as the aphasia filter is included in our approach.

Together, these components provide personalized predictions and interpretable localization of atypical speech behavior.

The main contributions of the current work are as follows:

1. Severity-Adaptive Transformer for Aphasia Keyword Classification (AQFormer). We introduce AQFormer, a transformer-based architecture that conditions intermediate speech representations on the speaker’s Aphasia Quotient (AQ) using Feature-wise Linear Modulation (FiLM). This design learns embedding from the aphasic speakers, resulting in improved classification performance for pathological language disorders compared to the baseline model, which treats all aphasic and non-aphasic speakers equally.
2. Aphasia-Specific Class Activation Mapping (A-CAM) Explanations with Dual-Stream. We propose a personalized aphasia method that results in dual complementary maps: one that supports keyword decision prediction by highlighting regions, and another that highlights atypical productions and disruptions associated with impairments. This design clearly differentiates between what reflects the disorder’s attributable flexibility and what supports the predicted category.
3. Impairment-informed constraints via a Multimodal Aphasia Filter (AF). We built an aphasia filter that brings together voice-activity signals, forced-alignment mismatches, and word-boundary deviations, all pulled into a single timeline mask. This mask goes straight into our attribution process. With this approach, our mask-consistent A-CAM sticks to non-negative, aphasia-aware relevance and zeroes in on regions that actually make linguistic sense. It cuts out those false positives in silent stretches that traditional CAM methods usually miss.
4. Stability of explanations under variability. We tackle speaker variability in aphasic speech by using an adaptive dual-filtering method. It combines gradient accumulation and segment-wise A-CAM aggregation over overlapping time windows. The result is stable, locally accurate heatmaps across the time-frequency plane. This approach makes our system much more robust than standard Grad-CAM versions.
5. Comprehensive Quantitative Evaluation on Aphasic Speech with Explanation Metrics. We used a keyword dataset from AphasiaBank to compare AQFormer + A-CAM with several strong baselines and different CAM variants. We did not just look at accuracy and F1-score, we also checked how good the explanations were. For that, we used deletion/insertion AUPC and ADCC and tested how well the explanations matched up with corpus annotations. (These annotations aren’t from clinicians; they are just a reference point.) In the end, our framework did not just beat the others at classification-it gave clearer, more focused explanations that lined up better with aphasia patterns.

## 2 Related work

### 2.1 Grad-CAM and its variants

XAI plays a vital role in research, especially when it comes to prediction and classification applications, where need to trust and understand how models arrive at their decisions, so transparency and interpretability matter a lot. Grad-CAM leads the way as a popular method; it offers visual insight into deep learning models by showing exactly which parts of an input drive the model’s predictions. This approach gives researchers and practitioners a clearer window into what the model actually “sees” and relies on. Several variants of Grad-CAM have been proposed to enhance its interpretability and applicability across different domains. For instance, Grad-CAM++ and Guided Grad-CAM (Chattopadhay et al., 2018), (Selvaraju et al., 2016) refine localization maps by accounting for each pixel’s importance and addressing issues with overlapping activations. Other variants, Integrated Grad-CAM, Augmented Grad-CAM, and Smooth Grad-CAM (Morbidelli et al., 2020; Sattarzadeh et al., 2021) have been developed to address the limitations of traditional Grad-CAM by enhancing CNN visual explanations through approaches such as score-based weighting, gradient integration, augmentation techniques, adherence to theoretical axioms, and noise reduction. Expected Grad-CAM aggregates Grad-CAM heatmaps from multiple layers to explain model predictions comprehensively (Buono et al., 2024). Extended Grad-CAM computes an averaged Grad-CAM by perturbing the input image multiple times, providing a probabilistic interpretation of the heatmap (Kim et al., 2023). A natural question is why a CAM-based approach, originally developed for image classification, is suitable for audio keyword classification. When processing speech as a time-frequency representation, such as a mel-spectrogram or feature maps from models such as WavLM, the classification task starts to look a lot like image classification, working with two axes: time and frequency or channel. CAM-based methods produce spatially coherent heatmaps that directly map onto these axes, yielding interpretable visualizations of which temporal and spectral regions drive the model’s decision. Methods like LIME require hundreds of model runs and rely on superpixels for explanations, but these rarely align with the actual acoustic units in speech. SHAP, on the other hand, gets computationally expensive fast, especially when dealing with deep models; plus, its attributes are sparse and assigned per feature, which does not fit speech well. Grad-CAM variants sidestep these headaches. They need just one forward and backward pass, so they are much faster. Their explanations align well with the model’s internal spatial architecture, and the resulting relevance maps look smooth and are much easier to interpret in speech applications. The approach simply fits.

A-CAM stands apart from earlier Grad-CAM extensions in a few big ways. First, it uses a dual-stream setup: one branch (a CNN) zeroes in on prediction-related details, while the other (a transformer paired with an aphasia filter) focuses on impairment-related clues. The older Grad-CAM techniques do not bother with separating out different kinds of information; they just dump one big relevance map and call it a day. But A-CAM takes things further. It brings clinical expertise into play with the aphasia filter, zeroing in on regions marked by odd pauses, phoneme errors, or unexpected boundary shifts. Instead of just crunching numbers, it tunes in to patterns clinicians notice and value. The third big change is how A-CAM handles disfluent speech, speech which is all over the place. Regular gradient methods get thrown off by messy timing and energy, but A-CAM uses an adaptive perturbation technique built for these quirks, keeping its results stable where others would stumble. A-CAM also does not just mash everything into a single global heatmap. Instead, it performs segment-wise aggregation, capturing all the local, phoneme-level ups and downs that a basic map would miss. Put all this together, A-CAM is not just a one-size-fits-all solution like Grad-CAM++ or Score-CAM. It directly tackles the messy, real-world challenges in interpreting pathological speech, thanks to its tight link with domain-specific knowledge and task-driven tweaks.

### 2.2 Explainable AI in audio classifications

Several studies have explored integrating explainability techniques with advanced deep learning models for audio classification. For instance, Kim et al. (2021) analyzed the classification of beehive sounds and visualization using Grad-CAM and Machine learning algorithms. Combined Grad-CAM with the VGG-13 model using MFCC as input data, which enhanced the performance and interpretability of the data and determined how the best-performing CNN model recognized audio scenes (Zhang et al., 2021). Incorporated Grad-CAM to evaluate what information is perceived by the CNN model. Acoustic scene classification based on the Mel spectrogram decomposition study utilized the CNN visualization method to generate statistics and perform analyses on the Mel spectrogram activation (Zhang et al., 2021). In the study (Cai et al., 2022) the authors combine the audio event detection task with the Grad-CAM algorithm. Several studies related to hate speech and emotion speech detection have utilized XAI techniques (Imbwaga et al., 2024; Mehta and Passi, 2022; Kibriya et al., 2024; Kim and Kwak, 2024) for analysis, specifically to evaluate the overall predictive performance of their models. Most of them integrated LIME, SHAP, and layer-wise relevance propagation (LRP), but were very limited in their use of Grad-CAM. Although Grad-CAM is primarily used for visual explanations in image classification to significantly improve the interpretability of CNN models, it is also being applied to other areas. Transformer-based models focus only on the impaired speech area; they do not go beyond that. In our study, we adapted the concept of CAM to analyze impaired speech, utilizing our improved explainability framework to address this gap. By integrating transformer-based modelling with clear, visual interpretations of aphasia-specific impairments, this work advances explainable, patient-adaptive AI in clinical speech assessment. By enabling both accurate keyword classification and clinically meaningful insights, the framework provides a foundation for personalized diagnosis and rehabilitation planning in aphasia.

When looking at explanation methods, one must draw a line between whether the explanation is true to how the model works (model faithfulness) and whether it is easy for clinicians to understand (clinical interpretability). Faithfulness metrics, such as deletion or insertion AUPC, assess whether the explanation aligns with the model’s logic and decision-making process. In other words, they test if the explanation reflects what’s going on in the model’s “mind.” In contrast, clinical interpretability addresses clinicians’ understanding, trust, and ability to take actionable steps based on explanations that enhance their clinical decision-making; it evaluates the connection between the explanation and the end user. These properties are logically independent: a faithful explanation can be clinically opaque, and a clinically plausible explanation can be unfaithful to the model’s actual reasoning. In this work, we look at A-CAM from three angles: (i) we test how faithful the model is using AUPC and ADCC metrics; basically, we want to see if A-CAM’s explanations show how the model makes its decisions. (ii) We check how well the system lines up with clinical standards. We do this by directly comparing the aphasia filter’s results to clinical labels, such as the severity of the condition or where phoneme-level errors occur (see Section 4.4). The results show that the filter’s behavior matches what experts already know in the clinical world, and (iii) we acknowledge that establishing full clinical interpretability ultimately requires human evaluation studies with speech-language pathologists, which represents an important direction for future work. The rest of the section is organized as follows. We introduced the proposed AQFormer with an Aphasia-specific CAM framework in Section III, presented the experimental results in Section IV, discussed and conducted an ablation study in Section V, and concluded in Section VI.

## 3 Methods

### 3.1 Proposed framework: AQFORMER with aphasia-specific CAM

#### 3.1.1 AQFormer: severity-adaptive transformer for aphasia keyword classification

The proposed model, AQFormer (Aphasia Quotient-aware Transformer for Spoken Keyword Classification), is a novel dual-stream architecture for robust spoken keyword classification. Takes raw audio waveforms as input, with shape (batch, samples), and passes them through a pre-trained WavLM-Large model, fine-tuning only the last four transformer layers. The WavLM encoder outputs a sequence of robust 1,024-dimensional feature vectors from the raw input utterance. The final four layers of the pretrained model have been fine-tuned. These features from the model are then passed through a lightweight, trainable 2-layer, task-specific Transformer head, which refines the sequence to produce a final 1,024-dimensional audio representation.

**Figure 1:**
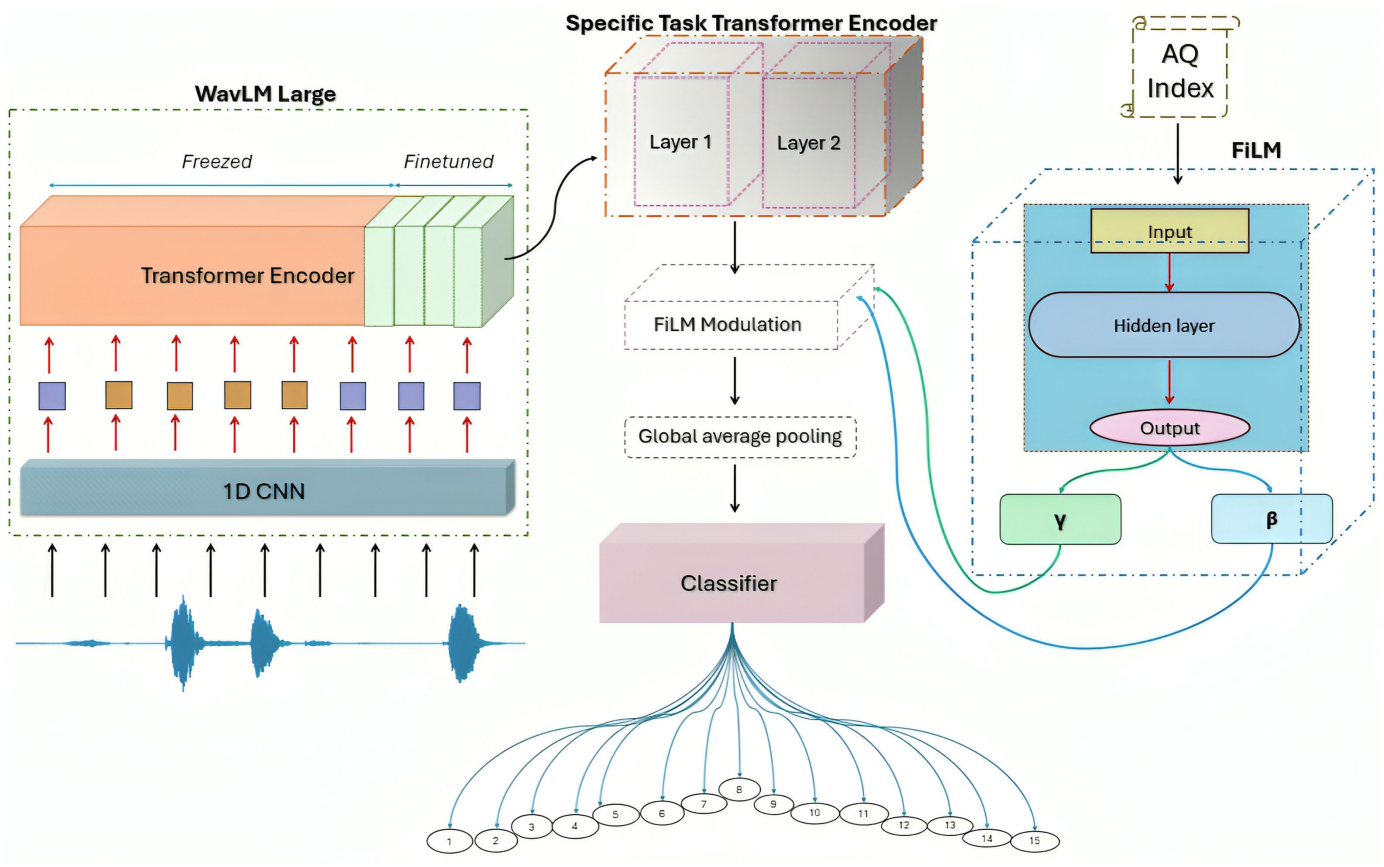
Proposed AQFormer architecture for spoken keyword classification.

In parallel, a secondary Feature-wise Linear Modulation (FiLM) generator MLP processes the AQ numerical index representing the target keyword class into a 2 x Hidden Dim vector. This vector is split into two learnable modulation vectors: gamma and beta, each with 1,024 dimensions. Using FiLM modulation, these vectors are broadcast across the time dimension to match the transformer output. Consequently, the audio features are modulated based on aphasia severity, producing AQ-conditioned features of shape (batch, timesteps, 1,024). To reduce the output to a fixed-size vector, a global average pooling operation is applied across the time axis. This vector is then fed into a Multi-Layer Perceptron (MLP) classification head, which produces the final logits for the 15 keyword classes. Unlike standard transformer keyword spotters that process only audio features, AQFormer’s FiLM modulation creates severity-adaptive representations. The gamma and beta vectors dynamically scale, and shift audio features based on the patient’s AQ score, enabling the model to adjust its internal processing to the severity of the aphasia. The AQ score is raw, unaltered continuous value, without normalization, feeding directly into the FiLM generator MLP. In our dataset, the AQ scores range from 20.5 to 94.8, with a mean of 71.0, spanning mild to severe impairment. The FiLM generator learns to translate the full range of clinical severity into beta and gamma modulation parameters, avoiding arbitrary imposed boundaries, using the raw score. Instead of ignoring severity, the model now accounts for it, which improves accuracy and makes interpretation clearer for personalized therapy. This marks a big shift. This setup lets the model respond directly to clinical needs. It’s much easier to understand what’s going on because the model adjusts its internal representation based on the actual severity of aphasia, leading to more accurate spoken keyword classification. The model architecture is illustrated in Figure 1.

#### 3.1.2 Aphasia-specific class activation mapping (A-CAM): dual interpretability framework

The Aphasia-specific Class Activation Mapping (A-CAM) framework tackles two big jobs: it points out which parts of a speech signal matter most for predicting the keyword, and it also highlights the regions linked to aphasia-related issues in spoken keywords. At the core of A-CAM is a special aphasia filter. This filter pulls together pause patterns, checks phoneme accuracy via forced alignment, and tracks any unusual timing shifts. It wraps these details into a structured temporal mask. When pair this mask with class-specific gradient information, A-CAM does not just highlight the evidence for its predictions; it brings mispronunciations, disfluencies, and boundary errors into focus, along with the main decision cues. The framework goes further than standard CAM by adding four key features. First, uses adaptive dynamic perturbation. Second, brings in dual-filtered gradient directional refinement, which helps lock down stable, positive relevance. Third is an explicit aphasia filter, ensuring impairment attributions stay within regions that make sense. Finally, applies segment-wise aggregation, giving the sharper, localized time-frequency explanations.

**Figure 2:**
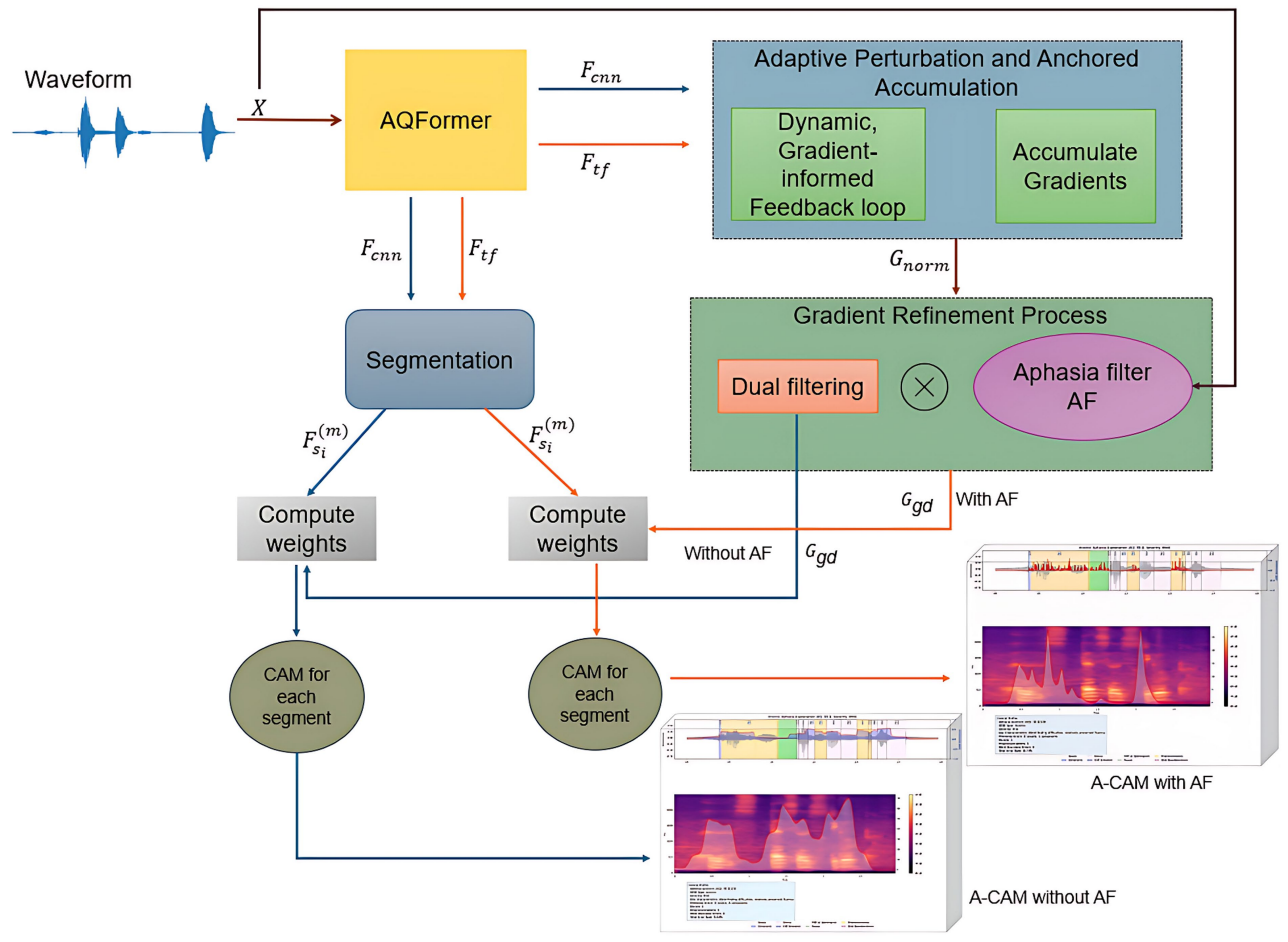
Overall framework of proposed AQFormer with aphasia-specific CAM.

A key component is the dual A-CAM architecture, which generates two complementary maps: (1) Transformer-layer A-CAM: works on WavLM transformer features and applies the aphasia filter to localize aphasia-specific error patterns (pauses, substitutions, boundary shifts) in the spectro-temporal domain. (2) CNN-layer A-CAM (without aphasia filter): operates on WavLM convolutional features to highlight regions most influential for the model’s keyword decision. Together, these streams provide model interpretability (what supports the prediction) and clinical interpretability (where atypical speech behavior occurs), forming a unified framework that links keyword classification with impairment-aware analysis (Figure 2).

##### 3.1.2.1 Feature representation

Given an input waveform X ∈ ℝ*L* of length *L,* the model extracts a feature map F from an intermediate layer *l* as (Equation 1)


$$F=\text{Mode}l_{l}(X)$$ (1)

where, F ∈ ℝ*T×K* represents the feature map, where *T* is the number of time steps after subsampling, and *K* is the number of channels. For the chosen CNN layer (prediction branch) with feature map F*cnn* ∈ ℝ*T’ × K* and the transformer layer (impairment branch) with feature map F*tf* ∈ ℝ*T” × K′.* Let $y^{c}$ denote the logit for the target class c . Gradients G for CNN and transformer feature maps with respect to the target class *c* are obtained as


$$G_{\mathrm{cnn}}=\frac{\partial y^{C}}{\partial F_{\mathrm{cnn}}},G_{\text{ft}}=\frac{\partial y^{C}}{\partial F_{\mathrm{tf}}}$$ (2)

##### 3.1.2.2 Adaptive perturbation and gradient accumulation

**Figure 3:**
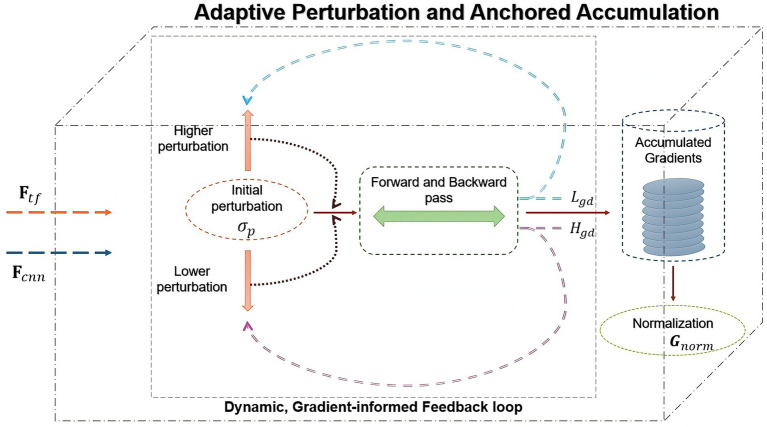
Adaptive perturbation and gradient accumulation.

To ensure robustness against variability in aphasic speech, an adaptive perturbation strategy dynamically adjusts the noise magnitude during back-propagation for each sample. Figure 3 shows the dynamic strategy. Initialize perturbation magnitude $\sigma_{p},$ for each perturbation, *p (where p = 1…N),* a scaled input $X_{\left(p\right)}=X+\sigma_{p}$ is generated, producing corresponding feature maps $F_{\mathrm{cnn}(p)}$ , $F_{\mathrm{tf}(p)}$ and gradients $G_{\mathrm{cnn}(p)}$ and $G_{\mathrm{tf}(P)}$ .
The dynamically accumulated gradients are computed as

$$G_{\mathrm{cnn}\text{dynamic}}=G_{\mathrm{cnn}}+\sum_{p=1}^{N}G_{\mathrm{cnn}(P)},G_{\mathrm{tf}\text{dynamic}}=G_{\mathrm{tf}}+\sum_{p=1}^{N}G_{\mathrm{tf}(P)}$$ (3)

The perturbation magnitude $\sigma_{p}^{\prime }$ is modified as Equation 4

$$\sigma_{p}^{\prime }=\min(P_{\max},\sigma_{p}+\text{step}\_\text{size}\times \text{mean}(|G_{\left(P\right)}|))$$ (4)

This process highlights consistent discriminative regions even in noisy or disfluent speech. Normalized gradients $G_{\mathrm{cnn}(\mathrm{norm})}$ and $G_{\mathrm{tf}(\mathrm{norm})}$ are obtained by


$$G_{\mathrm{cnn}(\mathrm{norm})}=\frac{G_{\mathrm{cnn}\text{dynamic}}}{\sqrt{\text{mean}({G_{\mathrm{cnn}}}_{\text{dynamic}}^{2})+1e^{-5}}},G_{\mathrm{tf}(\mathrm{norm})}=\frac{G_{\mathrm{tf}\text{dynamic}}}{\sqrt{\text{mean}({G_{\mathrm{tf}}}_{\text{dynamic}}^{2})+1e^{-5}}}$$ (5)

##### 3.1.2.3 Dual filtering of gradients

Gradients and feature maps of both branches are refined through a dual-filtering mechanism that retains only positive contributions, such as Equations 6a, 6b:


$$\begin{array}{l} G_{\mathrm{cnn}\mathrm{gd}}[t,k]=G_{\mathrm{cnn}\mathrm{norm}}[t,k]\times I(F_{\mathrm{cnnt},k\prime }>0)\\ \times I(G_{\mathrm{cnn}\mathrm{norm}}[t,k]>0) \end{array}$$ (6a)

$$G_{\mathrm{tf}\mathrm{gd}}[t,k^{\prime }]=G_{\mathrm{tf}\mathrm{norm}}[t,k^{\prime }]\times I(F_{\mathrm{tft},k\prime }>0)\times I(G_{\mathrm{tf}\mathrm{norm}}[t,k^{\prime }]>0)$$ (6b)

**Figure 4:**
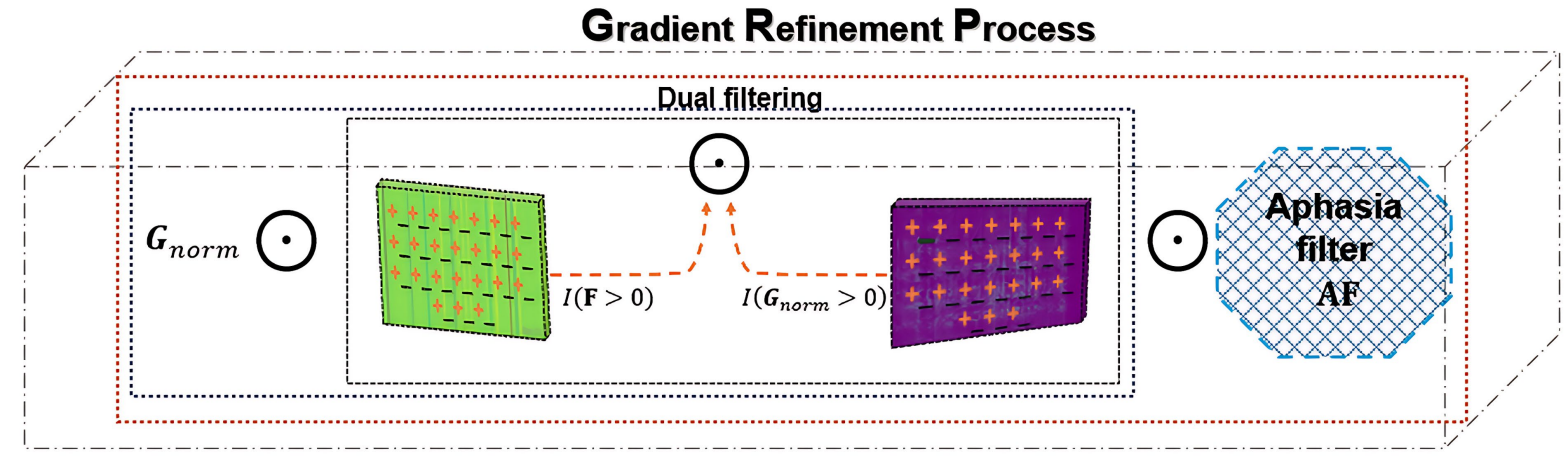
Dual filtering mechanism.

This selective operation ensures that the resulting CAM emphasizes features that positively influence the target class, reducing artefacts from irrelevant acoustic energy or background noise. The dual filtering mechanism is shown in Figure 4.

##### 3.1.2.4 Multimodal aphasia filter

**Figure 5:**
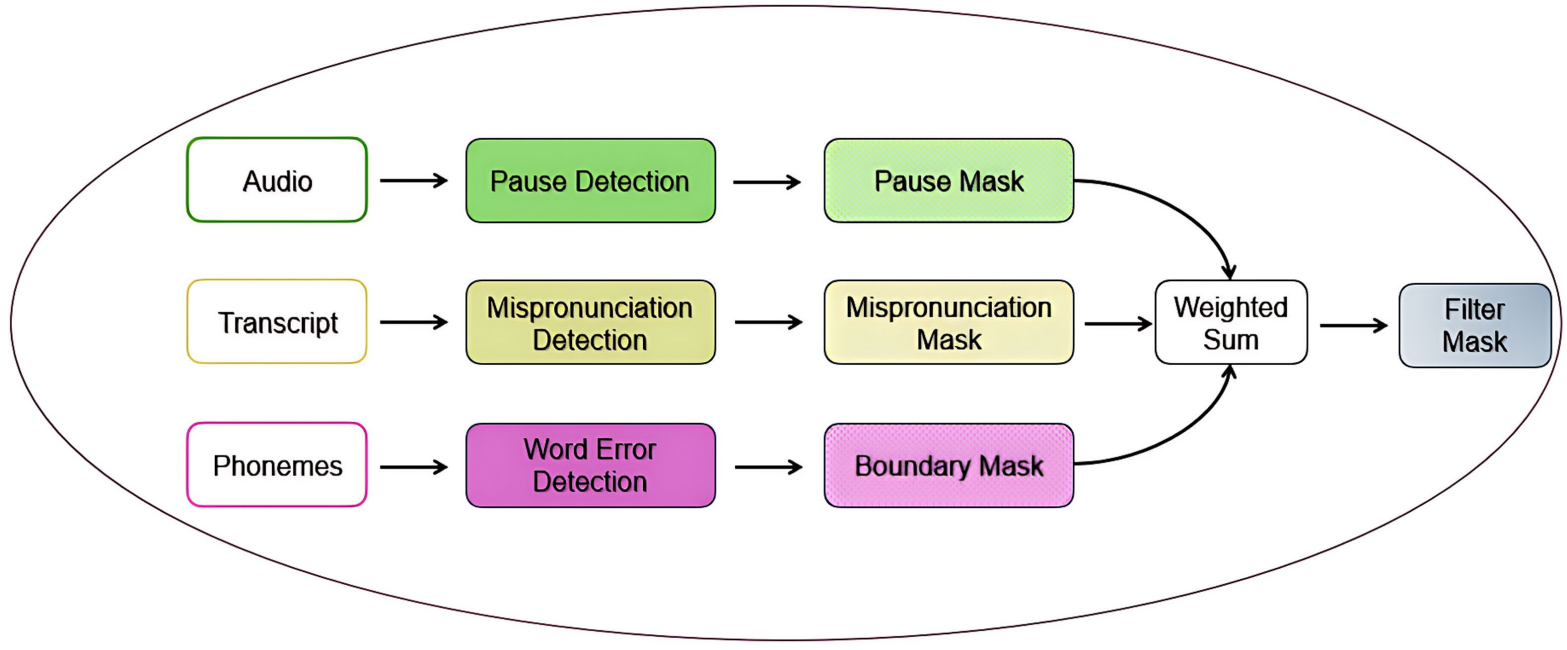
Aphasia filter workflow.

Aphasia filter (AF) is designed to highlight relevant regions in the feature map that correspond to aphasia-related speech patterns (pauses, mispronunciations, incorrect word boundaries). The aphasia filter workflow is shown in Figure 5.

Its primary role is to weight or mask the feature map, so the model focuses on the most critical regions associated with aphasia.

- *Pause Detection:* Voice-activity detection (VAD) partitions the waveform into speech and silence intervals; segments exceeding the threshold $T_{P}$ are labelled as pauses.
- *Phoneme Mispronunciation:* Forced alignment between the canonical phoneme sequence $Q_{c}$ and the produced sequence $Q_{\text{actual}}$ identifies substitution, insertion, or deletion operations; their temporal spans define mispronunciation intervals.
- *Word-Boundary Errors:* Temporal gaps between consecutive word segments beyond a threshold $T_{B}$ are flagged as boundary distortions.

For each anomaly type X ∈{Pause,,Mispronunciation,Boundary} , a binary mask $M_{X}(t)$ is created:


$$M_{X}[t]=\{\begin{array}{c} 1,t\;\epsilon \;\text{interval of}\;X\;\\ 0,\text{otherwise} \end{array}$$ (7)

A composite weighted mask is then defined as Equation 8:


$$AF_{W}^{1D}[t]=M_{P}^{1D}[t]\cdot \propto_{P}+M_{M}^{1D}[t]\cdot \propto_{M}+M_{\mathrm{WB}}^{1D}[t]\cdot \propto_{\mathrm{WB}}$$ (8)

where, $M_{P}^{1D}[t]$ , $M_{M}^{1D}[t]$ and $M_{\mathrm{WB}}^{1D}[t]$ are the binary masks for pauses, mispronunciations, and word boundary errors at the time frame t . $\propto_{P}$ , $\propto_{M}$ , $\propto_{W\;B}$ ≥ 0 are user-defined non-negative weights controlling the contribution of each anomaly type to the overall filter. For our experiments, we assigned the filter weights as follows: $\propto_{P}$ = 1.0, $\propto_{M}$ = 1.2, and $\propto_{W\;B}$ = 1.5. By doing this, we gave the most importance to disruptions at word boundaries. These disruptions really matter, since they point directly to difficulties in articulatory planning for people with aphasia. Next in importance are phoneme mismatches, which are more diagnostic than pauses, followed by a baseline weight for pauses, since they are common even in typical speech. These values were chosen based on empirical evidence from validation-set performance and the diagnostic relevance of each anomaly type.

**Figure 6:**
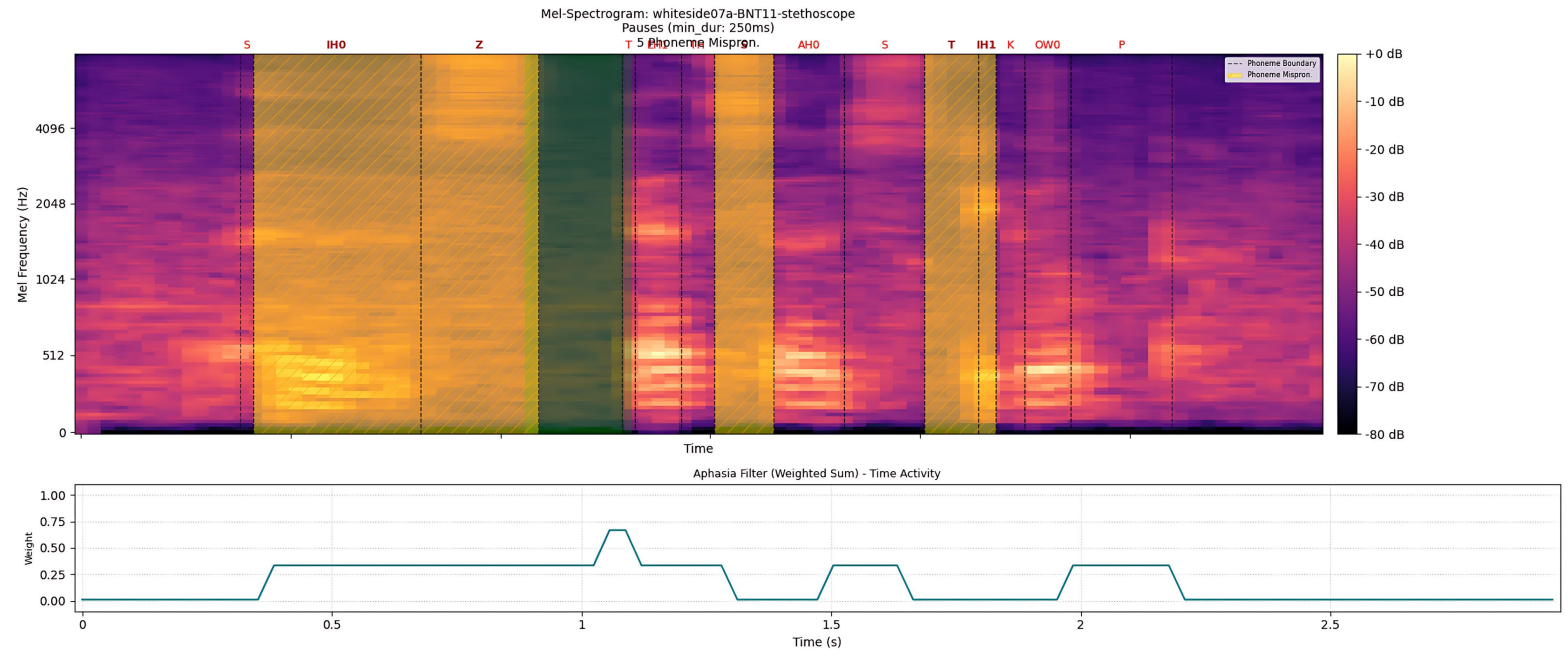
Aphasia filter result of aphasic spoken keyword ‘stethoscope’ with an aq value of 90.8.

**Figure 7:**
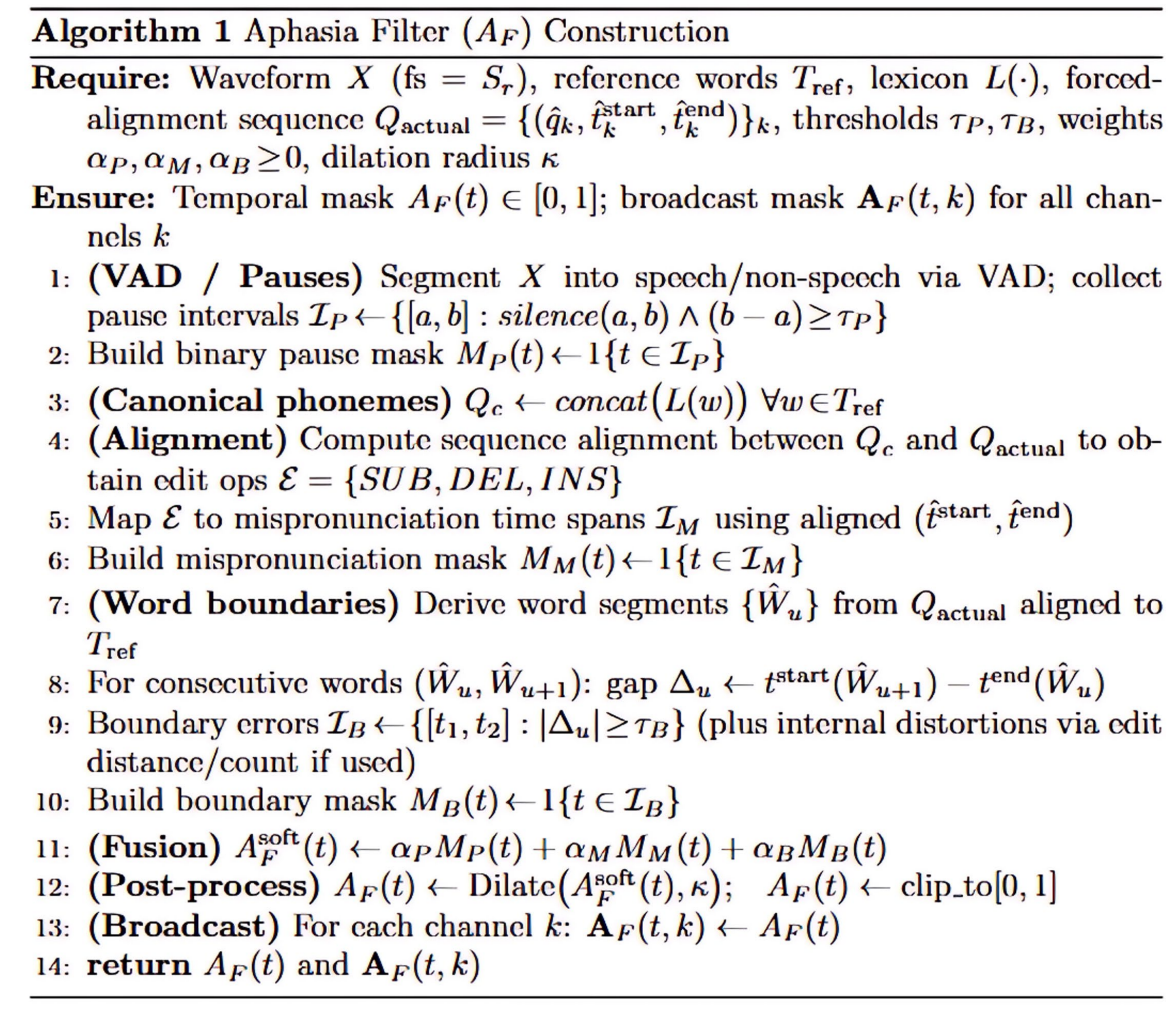
Algorithm for the construction of the aphasia filter.

The aphasia filter components were validated against AphasiaBank metadata transcriptions, which serve as clinical ground truth. Pause intervals, phoneme mispronunciations, and word boundary errors detected by our filter showed strong correspondence with clinician-annotated speech errors, confirming the filter’s clinical fidelity for highlighting diagnostically relevant regions. The example of the aphasia filter result for the keyword ‘stethoscope’ is shown in Figure 6. Figure 7 illustrates the algorithm used to construct the aphasia filter.

The aphasia filter is integrated into the transformer branch gradient refinement process, ensuring that only the most relevant features highlighted by the filter contribute to the final CAM. This gives a more targeted interpretation of the speech signal. The equation for the same is shown in Equation 9


$$\begin{array}{l} G_{\mathrm{tf}\mathrm{gd}}[t,k^{\prime }]=G_{\mathrm{tf}\mathrm{norm}}[t,k^{\prime }]\times I(F_{\mathrm{tft},k\prime }>0)\\ \times I(G_{\mathrm{tf}\mathrm{norm}}[t,k^{\prime }]>0)\times \mathrm{AF}[t] \end{array}$$ (9)

thereby gating relevance to clinically abnormal segments and generating error-localized explanations. Where, $G_{\mathrm{tf}\mathrm{gd}}[t,k^{\prime }]$ is the normalised gradient for channel *k’* at time *t*, $F_{\mathrm{tft},k\prime }$ is the original feature map for channel k’ at time *t,* $I(F_{\mathrm{tft},k\prime }>0)$ is an indicator function that retains positive activations in the feature map, $I(G_{\mathrm{tf}\mathrm{norm}}[t,k^{\prime }]>0)$ is an indicator function that retains positive gradients.

##### 3.1.2.5 Segment-wise CAM aggregation

**Figure 8:**
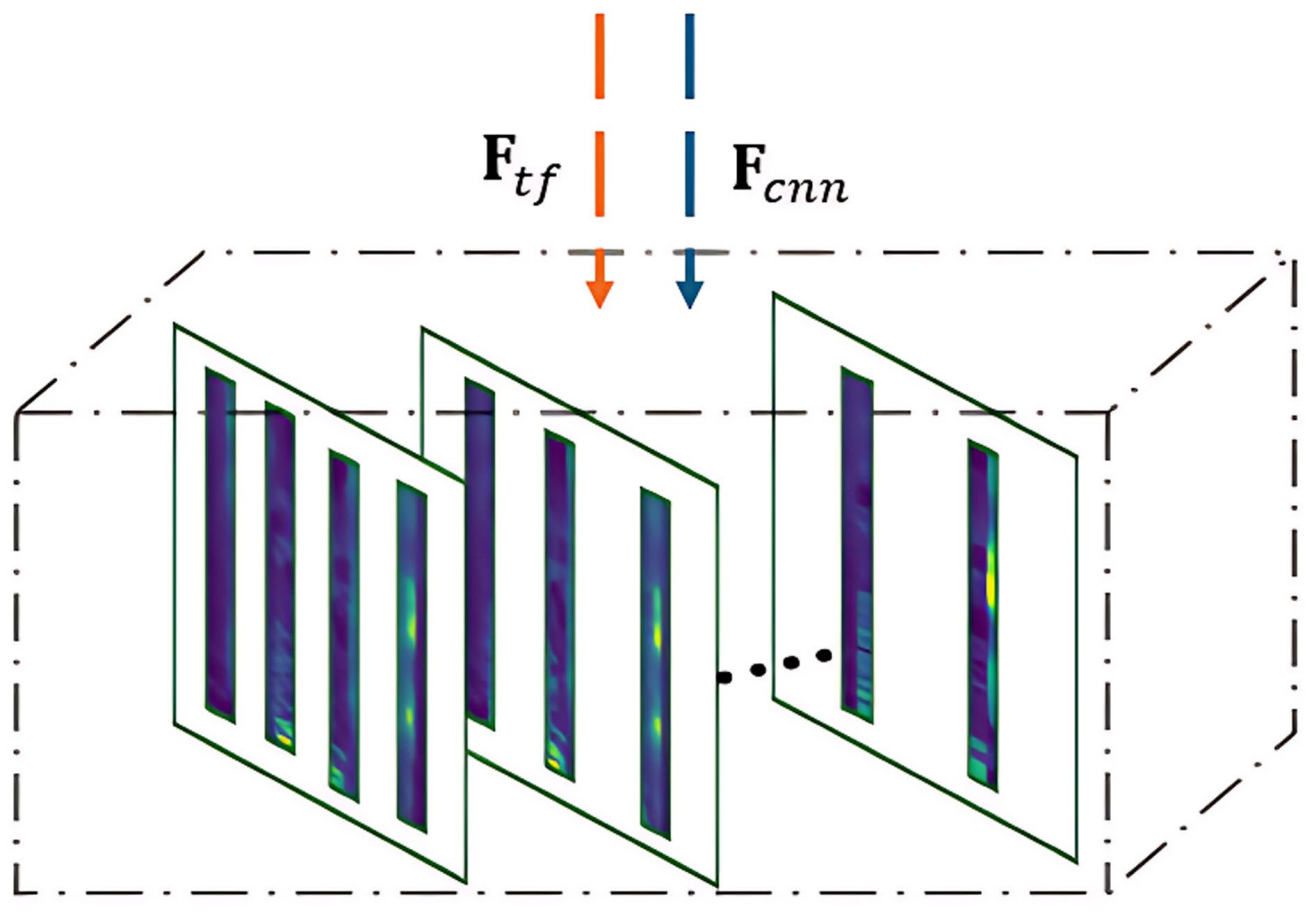
Segmentation of feature Map for CAM Aggregation.

To capture local phoneme-level variations, the framework independently segments the feature map of each A-CAM branch. Let $F_{\mathrm{cnn}}$ denote the CNN feature map used for the prediction-relevance A-CAM, and $F_{\mathrm{tf}}$ the Transformer feature map used for the aphasia-error A-CAM, as in Figure 8. Both feature maps are divided into overlapping temporal segments $S_{i}$ :


$$F_{s_{i}}^{\left(m\right)}=F_{s_{i}}[:,j_{m}:j_{m}+t_{\mathrm{seg}},:]$$ (10)

where, $F_{s_{i}}^{\left(m\right)}$ is the *m-th* segment of the feature map, $j_{m}\;$ is the starting index of the segment $j_{m}=m\times t_{\mathrm{seg}}\times (1-O)$ , m ∈ {0, 1…*M*-1} represents the segment index, *M* is the total number of segments, *O* denotes the overlap ratio, and $t_{\mathrm{seg}}$ is the length of the segment.

For each segment, compute the weights $\alpha_{k}^{s}[m]$ for each channel k, these weights represent the importance of each channel for segment *m* as given in Equations 11a, 11b:


$$\alpha_{k}^{\text{pred}}[m]=\frac{1}{t_{\mathrm{seg}}}\sum_{t=1}^{t_{\mathrm{seg}}}G_{\mathrm{cnn}\mathrm{gd}}[t,k]$$ (11a)

$$\alpha_{k}^{\text{impd}}[m]=\frac{1}{t_{\mathrm{seg}}}\sum_{t=1}^{t_{\mathrm{seg}}}G_{\mathrm{tf}\mathrm{gd}}[t,k]$$ (11b)

where, $\alpha_{k}^{s}[m]$ is the weight for channel *k* in segment *m.*

The segment-level class activation map is then obtained as Equations 12a, 12b:


$$\mathrm{CA}M_{m}^{\text{pred}}[t]=\sum_{k}\alpha_{k}^{s}[m]{F_{\mathrm{cnn}}}_{s_{i}}^{\left(m\right)}[t,k]$$ (12a)

$$\mathrm{CA}M_{m}^{\text{impd}}[t]=\sum_{k}\alpha_{k}^{s}[m]{F_{\mathrm{tf}}}_{s_{i}}^{\left(m\right)}[t,k^{\prime }]$$ (12b)

Here, $\mathrm{CA}M_{m}^{\text{pred}}[t]$ and $\mathrm{CA}M_{m}^{\text{impd}}[t]$ represent the localised relevance for segment m at time index t for the prediction and impairment views, respectively.

After Gaussian smoothing and resizing, segment maps are aggregated to form the final heatmap as Equations 13a, 13b:


$$A-\mathrm{CA}M_{\text{final}}^{\text{pred}}=\frac{1}{M}\sum_{m=1}^{M}\mathrm{CA}M_{m}^{\text{pred}}$$ (13a)

$$A-\mathrm{CA}M_{\text{final}}^{\text{imprd}}=\frac{1}{M}\sum_{m=1}^{M}\mathrm{CA}M_{m}^{\text{impd}}$$ (13b)

The final A-CAM (i) $A-\mathrm{CA}M_{\text{final}}^{\text{pred}}\;$ , computed without AF captures local spectro-temporal features driving the model’s decision, while (ii) $A-\mathrm{CA}M_{\text{final}}^{\text{imprd}}\;$ , computed with AF captures aphasia-related impairments in a temporally aligned manner.

## 4 Experiments and results

### 4.1 Experimental setup

All experiments were implemented in PyTorch and run in a Jupyter Notebook on an Ubuntu 22 workstation equipped with an NVIDIA RTX A5000 (64 GB) GPU. To ensure rigorous evaluation and prevent speaker leakage, we adopt speaker-disjoint splits: the 93 speakers were randomly divided into training (74), validation (8), and test (11) sets, ensuring that no speaker appears in more than one partition. As the datasets contain isolated spoken keywords, no additional segmentation was required. We employed various data augmentation techniques applied only to the training partition after splitting, including Gaussian noise, time stretching, and pitch shifting, to increase the dataset size and variability (Moëll et al., n.d.), resulting in 5138 samples. The validation and test sets include only original, unaugmented recordings: 4 in the validation set, 118 in the test set, respectively, so an unbiased estimation of generalization to unseen speakers can be obtained.

### 4.2 Data description

We employed an Aphasia dataset, a subset of the Aphasia Bank data (MacWhinney et al., 2011), containing recordings of aphasic patients performing 15 isolated spoken keywords (15 different output classes) from a total of 93 speakers with 960 samples. The dataset encompasses seven aphasia types: Anomic (33 speakers), Broca (23), Conduction (21), Wernicke (10), Transcortical Motor (3), Global (1), and two non-aphasic control speakers. Of these, 64 speakers (68.82%) present with fluent aphasia subtypes (Anomic, Conduction, Wernicke), and 27 speakers (29.03%) present with nonfluent subtypes (Broca, Transcortical Motor, Global). AQ scores range from 20.5 (severe) to 94.8 (mild), with a mean of 71.0. Speaker-disjoint splits were used as described in Section 4.1, with augmentation applied only to the training set.

### 4.3 AQFormer classification performance

**Figure 9:**
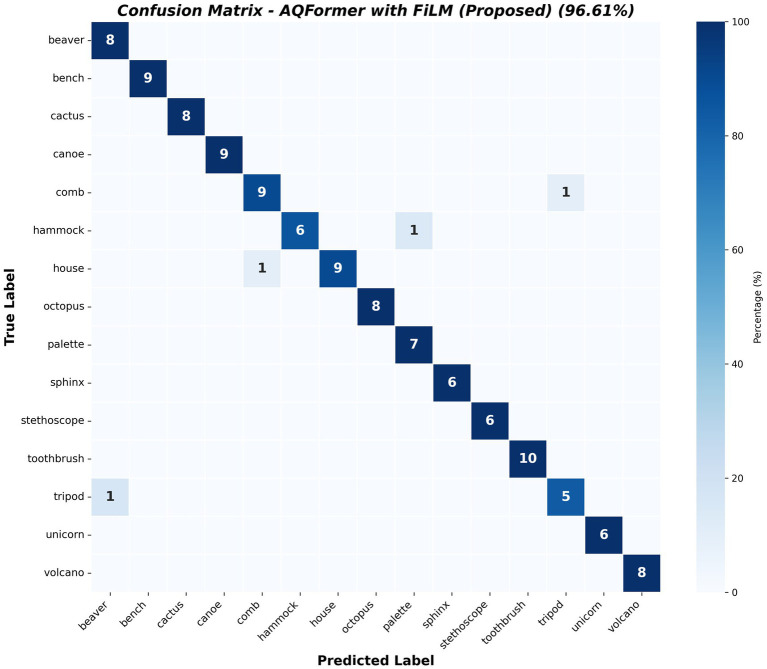
Confusion matrix of the proposed model.

The AQFormer is integrated into the A-CAM framework during the explainability phase to generate specific visual explanations. Table 1 presents the model’s performance metrics. The confusion matrix of the proposed model is shown in Figure 9. Table 2 shows the per-keyword performance breakdowns.

**Table 1:** Performance of AQFORMER for the aphasia dataset.

Model	Accuracy	Precision	Recall	F1-Score
Proposed model	96.61	95.2	96.6	96.8

**Table 2:** Per-keyword performance breakdowns.

Keyword	Support	Precision	Recall	F1
beaver	8	100.0	100.0	100.0
bench	9	90.0	100.0	94.7
cactus	8	100.0	100.0	100.0
canoe	9	100.0	100.0	100.0
comb	10	90.0	90.0	90.0
hammock	7	100.0	85.7	92.3
house	10	100.0	90.0	94.7
octopus	8	100.0	100.0	100.0
palette	7	87.5	100.0	93.3
sphinx	6	100.0	100.0	100.0
stethoscope	6	100.0	100.0	100.0
toothbrush	10	90.9	100.0	95.2
tripod	6	100.0	83.3	90.9
unicorn	6	100.0	100.0	100.0
volcano	8	100.0	100.0	100.0
Macro Avg	118	95.2	96.6	96.8

### 4.4 Quantitative validation of AF

To determine whether the aphasia filter genuinely detects meaningful linguistic errors rather than random variations in the sound spectrum, we conducted two quantitative tests: one assessed how well activations matched expected patterns, and the other evaluated how the filter’s activity correlates with phoneme boundaries from the Montreal Forced Aligner (MFA). Forced alignment used the MFA with a pre-trained English acoustic model. We acknowledge that in moderate-to-severe aphasia, phonological distortions and atypical productions reduce alignment accuracy. In our dataset, alignment was generally reliable for mild-to-moderate cases (AQ > 50, 75 of 93 speakers), where speech maintained enough phonological structure for the aligner. In severe cases (AQ < 50, 10 speakers), alignment errors could affect the aphasia filter, potentially introducing noise into the impairment-focused A-CAM. This issue is mitigated by the dual-filtering method, which only keeps positive gradient-activation pairs, and by segment-wise aggregation, which helps smooth out local alignment errors. Still, comparing alignment quality with manual annotations remains an important future step.

#### 4.4.1 Analysis 1: activation-level agreement

We computed the mean filter activation (averaged across 128 frequency bins and all-time frames) for each utterance, grouped by correctness label. Incorrectly produced utterances had a mean activation of 1.336, compared to 0.425 for correct utterances, a ratio of 3:1. A Mann–Whitney *U* test confirmed the difference was highly significant (*U* = 130,876, *p* < 1e-86, Cohen’s *d* = 2.05), indicating a large effect size. Table 3 shows the mean filter activation between correctly and incorrectly produced BNT utterances.

**Table 3:** Mean filter activation between correctly and incorrectly.

Metric	Value
Mean activation (correct)	0.425
Mean activation (incorrect)	1.336 (3.1 times higher)
Mann–Whitney U/*p*-value	130,876 / *p* < 1e-86
Cohen’s d (effect size)	2.05 (massive)
AQ correlation (speaker-level)	rho = −0.453, *p* < 1e-4

At the speaker level, mean filter activation was negatively correlated with the AQ, indicating that speakers with more severe aphasia elicited higher filter responses, with Spearman rho = −0.453 and *p* < 1e-4. At the frame level, filter activation showed a strong correlation with the number of phoneme mismatches in each utterance. With Spearman rho = 0.776, *p* < 1e-153, this confirms that the filter responds to articulatory deviations rather than general acoustic properties.

We applied the aphasia filter to all aphasia subtypes, and the results clearly showed that Global and Wernicke aphasia had the highest mean activation (1.15), followed by Conduction (0.92), TransMotor (0.89), Broca (0.80), and Anomic (0.77). This order matches what clinicians observe: posterior and global subtypes exhibit the most severe phonemic disruption, consistent with established severity hierarchies. Table 4 shows the mean filter activation across aphasia types.

**Table 4:** Mean filter activation across aphasia types.

Aphasia Type	Mean Activation (IoU)	Error Rate
Global	1.152	85.7%
Wernicke	1.155	55.4%
Conduction	0.923	55.2%
TransMotor	0.886	54.2%
Broca	0.798	49.0%
Anomic	0.768	38.3%

#### 4.4.2 Analysis 2: frame -level spatial overlap (IoU)

To evaluate whether the filter activates at the correct times, we calculated frame-level Intersection over Union (IoU) between the binarized filter mask (threshold = mean activation per utterance), and phoneme-level error regions derived from MFA TextGrid alignments across 758 BNT utterances.

Incorrectly produced utterances had a mean IoU of 0.461 (Dice = 0.62), significantly higher than the 0.314 IoU observed for correct utterances (Mann–Whitney *U*, *p* < 1e-22). This confirms that the filter not only responds more strongly to errors but also localizes them at the frame level. Per-keyword IoU for incorrect utterances ranged from 0.42 to 0.52, indicating consistent spatial accuracy across all 15 BNT items. Severity-stratified analysis showed increasing spatial overlap with aphasia severity: Moderate–Severe (AQ 26–50) yielded the highest mean IoU of 0.529, followed by Mild–Moderate (0.473) and Mild (0.424), indicating that the filter is most spatially particular for utterances with pronounced articulatory disruption.

### 4.5 Quantitative evaluation of A-CAM

We validated our method and other traditional CAM-based algorithms across all our tests. The final convolutional layer and the last transformer layer were selected for verification because they capture the model’s highest-level representations.

We evaluate explanation faithfulness using Deletion/Insertion Area Under the Perturbation Curve (AUPC) and holistic quality with ADCC; higher insertion AUPC and Average Drop–Coherency–Complexity ADCC, and lower deletion AUPC, indicate better explanations. These metrics are numerical values that can be measured and compared, reflecting the degree of change or improvement, making it easier to evaluate models across all classes and communicate results. They also assess how well the explanation aligns with the model’s actual decision-making process.

#### 4.5.1 Deletion and insertion metrics

The insertion, *Is,* and deletion, *Ds* metrics are commonly utilized to evaluate the reliability of a model’s explanation (Yoshikawa and Iwata, 2024). The insertion metric measures how rapidly the probability of a target label increases as the most significant regions identified by the explanation are progressively added to a baseline input. In contrast, the deletion metric assesses how rapidly the probability decreases as significant regions are incrementally removed from the original input. An explanation is deemed faithful if the insertion score is high and the deletion score is low, as this emphasizes regions that strongly influence the model’s prediction. The expressions for deletion and insertion metrics are:


$$\text{Insertion}=\frac{1}{K}\sum_{k=1}^{K}f_{c}(B⊙(1-M_{k})+x⊙M_{k})$$ (14)

$$\text{Deletion}=\frac{1}{K}\sum_{k=1}^{K}f_{c}(x⊙(1-M_{k})+B⊙M_{k})$$ (15)

where x is the original input, B is the baseline input, $M_{k}$ as the mask that reveals the top-k most important elements from the explanation and $f_{c}$ as the models predicted probability for the target class *c.*

#### 4.5.2 Area under the perturbation curve (AUPC)

The area under the deletion and insertion curves (AUC) is a quantitative metric used to assess the effectiveness of a CAM approach (Iyer et al., n.d.). For audio CAMs, the most relevant time–frequency bins identified by the CAM are ranked and gradually perturbed. In the deletion curve, the most important bins are removed first in fixed ratios, and the predicted class probability is recorded at each step. A steeper decline in probability indicates that the CAM has identified significant regions; therefore, a lower area under the deletion curve $d_{\text{aupc}}\;$ suggests a better explanation. Conversely, in the insertion curve, the least important bins are replaced with informative ones step by step, and the class probability is measured after each insertion. A strong CAM method yields a significant increase in probability, resulting in a higher AUC for insertion $i_{\text{aupc}}$ .

#### 4.5.3 Average drop–coherency–complexity (ADCC)

To provide a more holistic assessment of the interpretability of the proposed A-CAM framework in its baseline form (i.e., without the aphasia filter), we employed the ADCC metric (Iqbal et al., 2024). Unlike conventional single aspect measures such as deletion or insertion, ADCC integrates three complementary properties of an ideal explanation map: *Coherency*, Stability of the CAM when conditioned on itself. *Complexity*, Sparsity of the highlighted regions, measured with the L1 norm. *Confidence Drop*, Reduction in model confidence when relying on the CAM. The three components are combined into a single metric through the harmonic mean:


$$\text{ADCC}(x)={\left(\frac{1}{3}[\begin{array}{l} \frac{1}{\text{Coherency}(x)}+\frac{1}{1-\text{Complexity}(x)}\\ +\frac{1}{1-\text{AverageDrop}(x)} \end{array}]\right)}^{-1}$$ (16)

where *Coherency(x)*, *Complexity(x)*, and *AverageDrop(x)* are normalized between 0 and 1. Higher ADCC values indicate explanation maps that are simultaneously coherent, compact, and reliable.

Table 5 compares quantitative metric scores across Grad-CAM variants. In our experiments, A-CAM without the aphasia filter achieves consistently higher ADCC scores than Grad-CAM, Guided Grad-CAM, and Integrated Grad-CAM, indicating more stable and compact explanations. For deletion/insertion AUPC, A-CAM yields lower deletion AUPC and higher competitive or improved insertion AUPC than existing CAM variants, demonstrating greater faithfulness to the model’s decision process.

**Table 5:** Comparison of qualitative metric scores with Grad-CAM variants.

XAI variants	Metrics						
	*Ds* ↓	*Is* ↑	AUPC		*Coherence ↑*	*Complexity ↓*	ADCC ↑
			$d_{\text{aupc}}$	$i_{\text{aupc}}$			
Grad-CAM	0.48	0.69	0.47	0.67	0.84 ± 0.20	0.93 ± 0.05	0.69 ± 0.16
Guided Grad-CAM	0.54	0.63	0.53	0.62	0.82 ± 0.16	0.95 ± 0.03	0.68 ± 0.15
Integrated Grad-CAM	0.39	0.74	0.38	0.72	0.81 ± 0.22	0.84 ± 0.11	0.70 ± 0.15
Proposed method	**0.38**	**0.73**	**0.37**	**0.71**	**0.85 ± 0.19**	**0.75 ± 0.11**	**0.71 ± 0.14**

When the aphasia filter is enabled, the impairment-focused A-CAM further concentrates relevance on clinically plausible regions (pauses, phoneme mismatches, boundary errors) without degrading predictive faithfulness, providing a targeted view of aphasia-related deviations.

#### 4.5.4 Impairment-focused A-CAM attribution agreement

The preceding analyses validated the aphasia filter in isolation. To confirm that filter-derived error patterns propagate to the final impairment-focused A-CAM attribution maps, we analyzed the A-CAM outputs (impairment branch, which highlights pronunciation error regions) across 685 matched BNT utterances, with the refined A-CAM (after aphasia filter gating). The quantitative agreement metric score for A-CAM with the aphasia filter is shown in Table 6.

**Table 6:** Quantitative agreement metric for A-CAM with AF.

Metric	Refined A-CAM (with AF)
Activation ratio (incorr/corr)	3.30x
Cohen’s *d*	1.61
AQ severity correlation (rho)	−0.754
IoU incorrect vs. error regions	0.807
|IoU correct vs. error regions	0.393

The impairment-focused A-CAM achieved a mean IoU of 0.807 (Dice = 0.878) for incorrectly produced utterances, significantly higher than the 0.393 observed for correct productions (Mann–Whitney U, *p* < 1e-67). This represents a 72% improvement over the base CAM IoU of 0.470 for incorrect utterances, demonstrating that the aphasia filter substantially improves spatial alignment between the attribution maps and actual error regions. Per-keyword IoU for incorrect utterances ranged from 0.68 to 0.89, confirming consistent spatial accuracy across all 15 BNT items.

At the speaker level, mean A-CAM activation correlated with clinical severity at Spearman rho = −0.754 (p < 1e-14) after filter gating, compared to rho = −0.374 (*p* = 0.001) for the base CAM, a doubling of the correlation strength. Severity-stratified IoU confirmed that the impairment-focused A-CAM is most spatially precise for severe cases: Severe (AQ 0–25) = 0.896, Moderate–Severe (AQ 26–50) = 0.887, Mild–Moderate = 0.801, Mild = 0.787.

Per-aphasia-type analysis confirmed clinically consistent ordering: Global (0.049) > Wernicke (0.040) > Broca/TransMotor (0.033) > Conduction (0.029) > Anomic (0.014), with the non-aphasic control speakers showing appropriately low activation (0.012).

#### 4.5.5 Qualitative analysis of A-CAM with AF

**Figure 10:**
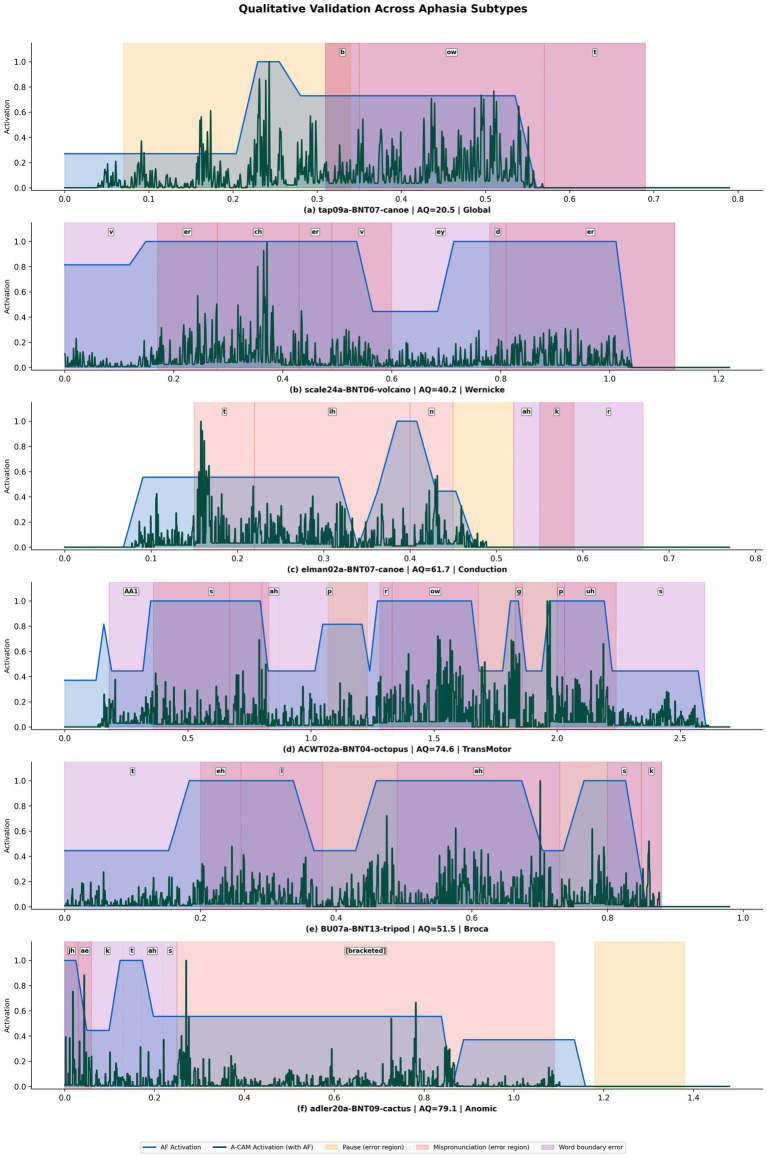
Qualitative validation of AF and A-CAM alignment against phoneme-derived error regions.

Figure 10 presents qualitative validation of AF and A-CAM alignment against phoneme-derived error regions across all six WAB aphasia subtypes, ordered by decreasing clinical severity. The Aphasia Filter activation (blue) consistently elevates within annotated error regions, mispronunciation zones (red shading), abnormal pauses (orange), and word boundary deviations (purple) while remaining attenuated in correctly produced segments. The A-CAM activation (teal), computed from Transformer features modulated by the AF, further refines this localization by concentrating activation at specific temporal frames within error regions.

For the Global aphasia speaker (a; AQ = 20.5, “canoe”), both AF and A-CAM activations span nearly the entire utterance, reflecting the pervasive phonological disruption characteristic of the most severe impairment class, with sustained pause and mispronunciation errors dominating the signal. For the Wernicke’s aphasia speaker (b; AQ = 40.2, “volcano” produced as “v-er-ch-er-v-ey-d-er”), AF and A-CAM activations concentrate almost exclusively within error regions with near-zero activation in error-free intervals, capturing the phonemic paraphasia typical of fluent aphasia despite the absence of effortful production patterns. The Conduction aphasia speaker (c; AQ = 61.7, “canoe” produced as “t-ih-n-ah-k-r”) exhibits intermittent mispronunciation clusters separated by fluent segments, with both AF and A-CAM tracking these discrete error bursts while diminishing in correctly produced intervals. For Transcortical Motor aphasia (d; AQ = 74.6, “octopus”), AF and A-CAM selectively peak at dispersed word boundary deviations and mispronunciation regions across a longer utterance while remaining attenuated elsewhere. The Broca’s aphasia speaker (e; AQ = 51.5, “tripod” produced as “t-eh-l.ah.s-k”) shows the nonfluent pattern: AF captures prolonged hesitations as sustained high-activation plateaus, while A-CAM concentrates on mispronunciation and boundary error segments. Finally, the Anomic aphasia speaker (f; AQ = 79.1, “cactus”) shows the sparsest activation pattern, with AF and A-CAM responding only to localized errors in the initial portion, while remaining low elsewhere, consistent with the primarily lexical-semantic nature of anomic errors that the acoustic-phonological AF captures only partially.

### 4.6 Dual A-CAM visualization

**Figure 11:**
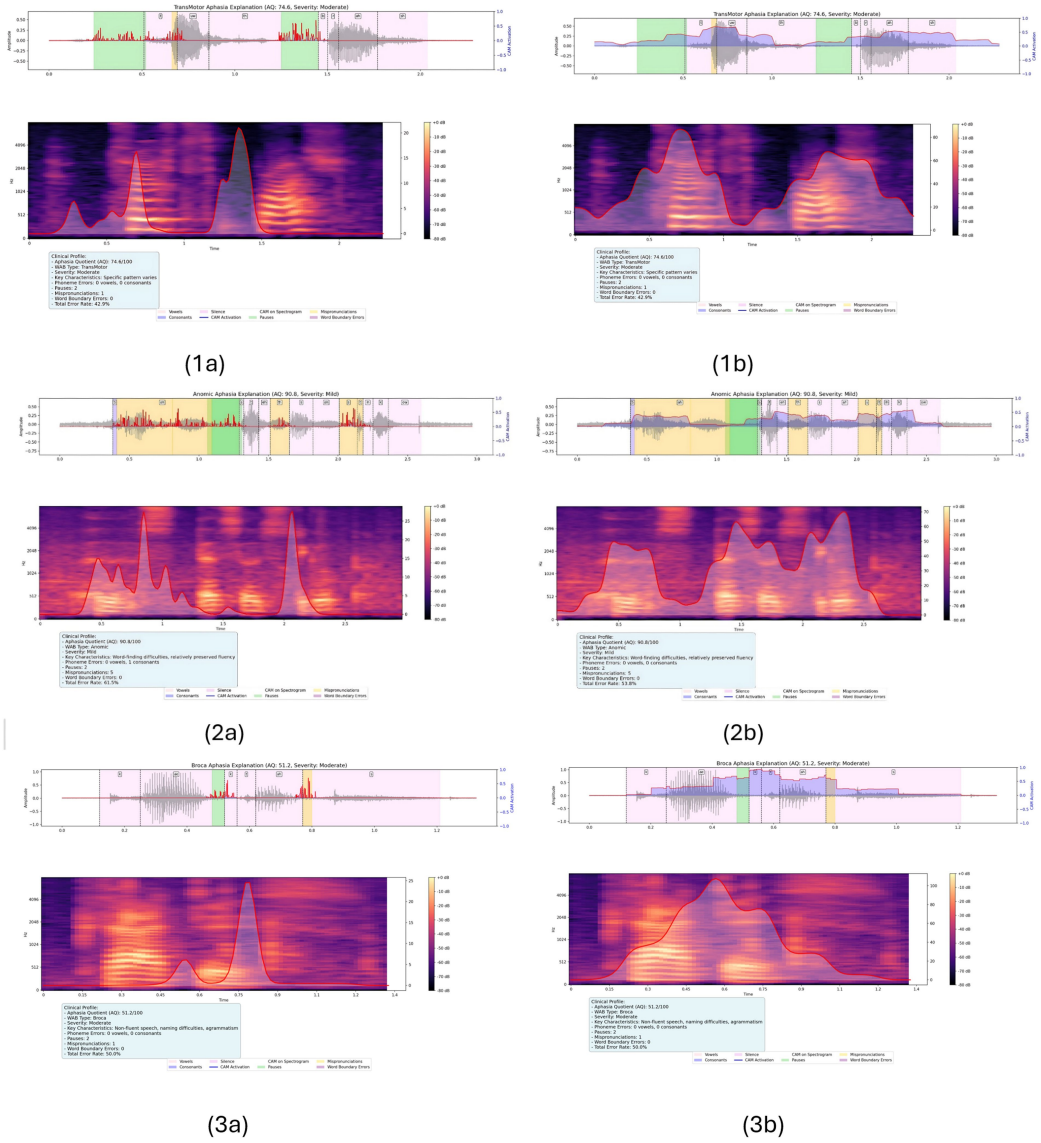
A-CAM, with and without the aphasia filter, shows the aphasia error patterns and the region highlighted by the model’s decision. **(1a)** Moderate level TransMotor aphasia with aq value 74.6 for the keyword “toothbrush” with the aphasia filter. **(1b)** Moderate level TransMotor aphasia with aq value 74.6 for the keyword “toothbrush” without the aphasia filter. **(2a)** Mild level Anomic aphasia with aq value 90.8 for the keyword “stethoscope” with aphasia filter. **(2b)** Mild level Anomic aphasia with aq value 90.8 for the keyword “stethoscope” without aphasia filter. **(3a)** Moderate level Broca aphasia with aq value 51.2 for the keyword “cactus” with aphasia filter. **(3b)** Moderate level Broca aphasia with aq value 51.2 for the keyword “cactus” without aphasia filter.

To demonstrate the complementary nature of our dual A-CAM approach, Figure 11 presents the comparisons: (1) *Left panels:* Transformer-layer A-CAM with aphasia filter, revealing raw aphasia error patterns in mel-spectrogram representations. (2) *Right panels:* CNN-layer A-CAM without aphasia filter, highlighting regions that contribute to correct keyword classification while emphasizing clinically relevant speech segments. This dual visualization, shown as Figure 11, enables clinicians to simultaneously understand (a) where specific aphasia-related speech impairments (pauses, phonological errors, boundary issues) manifest in the patient’s production, and (b) what acoustic features drive the AI’s classification decision. The transformer-layer A-CAM provides assessment-supporting insights, while the CNN-layer A-CAM enhances model transparency.

## 5 Discussion

The proposed framework combines three tightly coupled elements: severity-aware modelling, dual-stream attribution, and aphasia-informed filtering, to advance interpretable learning for pathological speech. First, AQFormer introduces explicit clinical-AI integration by conditioning internal representations on Aphasia Quotient (AQ) via FiLM, enabling the model to adjust its feature space according to impairment severity rather than treating all speakers uniformly. Second, the dual A-CAM design decouples prediction-focused relevance (CNN branch) from impairment-focused relevance (transformer branch), offering complementary views of what drives the decision and where atypical behavior occurs. Third, the multimodal aphasia filter injects clinically motivated cues, pauses, phoneme mismatches, and boundary deviations directly into the attribution process, constraining impairment maps to regions consistent with known aphasia patterns. Together, these components move beyond generic Grad-CAM adaptations and provide a task- and disorder-specific interpretability framework.

### 5.1 Ablation study

The ablation study of the final model architecture for the spoken keyword classifier quantitatively validates each architectural innovation: (1) AQ-index integration improves accuracy by 4%, confirming clinical data’s predictive value; (2) task-specific transformer encoder adds 3.1%, demonstrating domain adaptation benefits; (3) FiLM modulation contributes 2.26%, validating severity-adaptive feature modulation; (4) WavLM fine-tuning adds 1.69%, showing aphasia-specific acoustic pattern learning. The cumulative 11.05% improvement over the base model establishes that our integrated clinical-AI approach substantially outperforms standard audio-only classification. Table 7 shows the results of the ablation study conducted on the model architecture.

**Table 7:** Ablation study on architecture.

Model	Input	Test Accuracy
WavLM(finetuned) + MLP (base model)	Audio	86.03
WavLM(finetuned) + MLP (base model)	Audio + AQ index	90
WavLM + Task-specific encoder + MLP	Audio + AQ index	91.48
WavLM + Task-specific encoder + FiLM + MLP	Audio + AQ index	94.10
WavLM (finetuned) + Task-specific encoder + FiLM + MLP (Proposed Model)	Audio + AQ index	96.61

**Figure 12:**
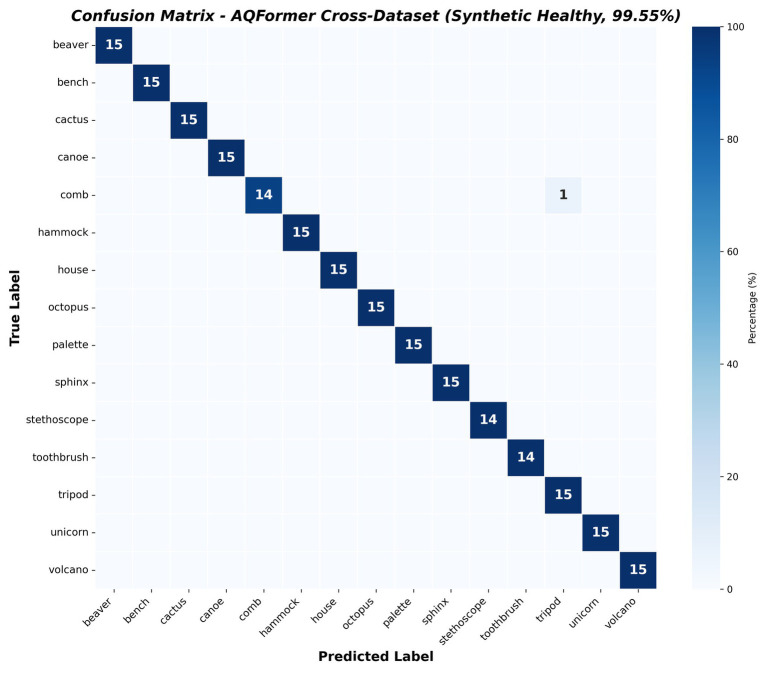
Confusion matrix of the cross-validated data.

To assess whether the model’s keyword representations generalize beyond aphasic speech, we tested the trained model on a new dataset. As none of the existing public speech datasets contain BNT keyword utterances, we created synthetic utterances from 15 speakers, totaling 223 samples. The generated data covers all 15 keywords, and none of these is included for training. The AQ score is set as 93.8 to simulate healthy minimum FiLM modulation. The model achieves 99.55% accuracy, with 13 of 15 keywords achieving perfect precision and recall. In 222 out of 223 cases, a single misclassification for one speaker, with a comb instead of a tripod, yields a macro F1 of 0.9956. Compared with the aphasic test set (96.61%), performance on healthy speech is expected to improve. This confirms that the model learned strong acoustic-phonetic keyword representations instead of aphasia-specific artefacts. With a high AQ score, FiLM conditioning minimizes severity-dependent modulation, letting the classifier rely on clean features. The performance metric score in Table 8 and the confusion matrix in Figure 12.

**Table 8:** Cross dataset validation using Synthetic dataset.

Model	Accuracy	Precision	Recall	F1-score
Proposed model	99.55	99.58	99.56	99.568

Ablations on the A-CAM components (Table 9) further validate the explainability design. Removing dual filtering results in a degradation of almost 5%, indicating reduced faithfulness. Excluding the dynamic perturbation strategy resulted in approximately a 6% decrease in performance, indicating reduced robustness to variability in disfluent speech. These trends support the need for guided, perturbation-stabilized, and segment-aware attribution in the pathological speech setting, where naïve CAM variants are sensitive to spurious energy and irregular timing.

**Table 9:** Ablation study on aphasia specific CAM.

Excluding elements	Metrics			
	Ds ↓	Is ↑	AUPC	
			$d_{\text{aupc}}$	$i_{\text{aupc}}$
Dual filtering	. 0.39	. 0.71	0.38	0.68
Dynamic perturbation	. 0.44	. 0.67	0.43	0.65

To evaluate whether AF and A-CAM capture meaningful error structure beyond simpler alternatives, we compare against four baselines (Table 10): a Random baseline (temporally shuffled AF activations, averaged over 50 trials), an Energy baseline (RMS amplitude), an Inverted Energy baseline (1 − RMS, naive pause detector), and the Base CAM without AF modulation. All methods are binarized using per-sample Otsu thresholding, with IoU computed against phoneme-derived error regions across the utterances. The Energy (0.300) and Inverted Energy (0.324) baselines show that acoustic amplitude alone provides limited error localization, while the Base CAM achieves the lowest IoU (0.150), confirming that standard Transformer attributions do not inherently target error regions. In contrast, the Aphasia Filter achieves 0.472 IoU (+57% over the best energy baseline), and the Refined A-CAM reaches 0.721, a 53% improvement over AF alone and 4.8 times the Base CAM, confirming that AF conditioning effectively steers attention towards impairment-relevant regions.

**Table 10:** Ablation study of error localization components against phoneme-derived error regions.

Method	IoU ↑	DICE ↑
Random baseline	0.405	0.534
Energy (RMS)	0.300	0.412
Inv. Energy (1 − RMS)	0.324	0.456
Base CAM (no AF)	0.150	0.256
Aphasia filter (ours)	**0.472**	**0.585**
Refined A-CAM (ours)	**0.721**	**0.821**

### 5.2 Effect of the aphasia filter

The aphasia filter specifically targets clinical interpretability. Without the filter, A-CAM highlights regions important for classification, but sometimes assigns relevance to nonspecific pauses or boundary artifacts. With the filter enabled, the impairment-focused A-CAM focuses on segments exhibiting phonological distortions, atypical pauses, and misaligned word boundaries, using *AphasiaBank/TalkBank* metadata as a silver-standard reference. This leads to error-localized maps that better align with aphasia characteristics, while the prediction-focused branch continues to capture discriminative keyword evidence. Occasional activation over leading or trailing silences is recognized as a limitation and can be excluded during expert interpretation.

Across the three aphasia profiles in Figures 11(1a–3a), the effect of the aphasia filter is consistent with type-linked production characteristics, which helps explain why the filtered maps differ from the unfiltered baseline. For the speaker with Transcortical Motor aphasia (moderate AQ), nonfluent output and initiation difficulties commonly manifest as pause-heavy production and disrupted temporal organization; accordingly, the filtered A-CAM tends to down-weight nonspecific silence while concentrating relevance on segments where boundary timing and mismatch cues co-occur with acoustic evidence for the target keyword. For Anomic aphasia (mild AQ), production is often relatively fluent but dominated by lexical retrieval difficulty, which may yield brief hesitations with comparatively fewer sustained phonological deviations; in this case, the filter places less emphasis on widespread mismatch regions and retains attribution primarily over compact discriminative segments while suppressing incidental pause intervals. In Broca’s aphasia (moderate AQ), nonfluent speech is frequently accompanied by phonological/phonetic distortions and instability in segmentation, leading to stronger or more spatially extended mismatch- and boundary-related cues; correspondingly, the filtered maps concentrate attribution in those impairment-linked regions while reducing spurious relevance to silence. These observations should be interpreted as qualitative alignment with known syndrome-level tendencies at the keyword level, rather than as diagnostic signatures of aphasia type. Figures 11(1b–3b) shows baseline A-CAM without the aphasia filter, which correctly emphasizes regions supporting keyword classification but occasionally attributes importance to nonspecific pauses. This behavior is noted as a limitation motivating the aphasia-filtered variant.

Regarding the applicability of the aphasia filter across aphasia subtypes, we note that the filter components (pause detection, phoneme mismatch, boundary deviations) were applied uniformly across all aphasia types in our dataset. While these features are most characteristic of nonfluent aphasia subtypes, our dataset comprises predominantly fluent speakers (52/74, 70%). Quantitative subtype-stratified analysis (Section 4.4.1) reveals that mean filter activation follows a clinically consistent ordering: Global (1.15) and Wernicke (1.16) show the highest activation, followed by Conduction (0.92), Transcortical Motor (0.89), Broca (0.80), and Anomic (0.77). Critically, this ordering demonstrates that the filter captures meaningful variation within the fluent subtypes themselves: Wernicke aphasia (a fluent subtype with posterior lesions) shows 1.5x higher activation than Anomic aphasia (the mildest fluent subtype), reflecting the greater degree of phonemic disruption in Wernicke’s aphasia despite its fluent presentation. This suggests that the filter responds to production-level error patterns across the fluent-nonfluent spectrum rather than solely to nonfluent temporal disruptions. The relative diagnostic contribution of each filter component may nonetheless differ across subtypes; for instance, pause detection may be more informative for Broca’s aphasia, while phoneme mismatch may be more relevant for Conduction aphasia. A fine-grained analysis of individual filter-component contributions per subtype is a promising direction for future work.

### 5.3 Role of aphasia quotient conditioning

A relevant question is whether the AQ score serves as a genuine severity signal that enables adaptive processing across impairment levels, or whether it instead acts as speaker-level metadata, providing auxiliary information correlated with speaker identity. In our dataset of 93 speakers, AQ scores range from 20.5 to 94.8, and multiple speakers share similar AQ values within each severity band (e.g., 33 speakers fall in the mild range with AQ > = 76). If AQ merely encoded speaker identity, the model would need to memorize individual AQ-speaker mappings, which would not generalize to unseen speakers at test time. The speaker-disjoint evaluation protocol ensures that test speakers were never seen during training; therefore, the FiLM modulation must generalize based on the AQ value itself rather than any learned speaker association. This is further supported by the strong correlation between AF activation intensity and AQ scores (*ρ* = −0.754, *p* < 0.001), indicating that the FiLM-conditioned representations systematically modulate with clinical severity rather than individual speaker characteristics. The raw AQ score was fed directly to the FiLM generator without normalization, allowing the network to learn its own mapping from the continuous clinical scale to modulation parameters.

### 5.4 Limitations

Several limitations should be considered when interpreting the results of this study. First, the experimental paradigm is restricted to isolated word-level productions from the Boston Naming Test and does not encompass sentence-level, discourse-level, or interactive speech contexts, which are ecologically more variable and clinically more complex. Extending this framework to continuous speech, where one must identify keyword boundaries and classify them, seems logical and represents the next challenge. Regarding the aphasia filter, it effectively detects error patterns across both fluent types, such as Wernicke and Conduction aphasia, and nonfluent types, like Broca and Global aphasia, aligning with the clinical severity order described in Section 4.4.1. However, it has difficulty with Anomic aphasia, showing the lowest A-CAM activation at 0.014, indicating limited sensitivity in that area. This is expected clinically, as Anomic errors are mostly semantic (word-finding failures, tip-of-the-tongue phenomena) rather than acoustic (phonemic distortions, pauses, boundary deviations), which are what the filter is designed to detect. Incorporating semantic-level features such as naming latency, hesitation patterns, or lexical-semantic embedding could improve the detection of Anomic error patterns, marking an important direction for future research. The evaluation was based solely on one dataset, AphasiaBank. We have yet to test whether the model generalizes across different datasets, especially non- English aphasic speech, which requires further investigation. Forced alignment with MFA tends to struggle with severe aphasia, and we did not assess alignment reliability independently. For explanation quality, model-centric faithfulness metrics such as AUPC and ADCC were employed. These metrics show that A-CAM explanations remain faithful to the model’s reasoning, but they do not demonstrate clinical utility. Further clinician-focused evaluation studies are necessary to fully assess the practical value of these explanations. We have bolstered validation of the aphasia filter through quantitative activation-level agreement (Cohen’s *d* = 2.05) and frame-level IoU analysis against MFA phoneme boundaries (IoU = 0.461 for incorrect utterances), providing convergent evidence that the filter captures meaningful error patterns. Nonetheless, human evaluation by speech-language pathologists remains essential to verify interpretability claims and determine whether the highlighted regions correspond to clinically actionable production features.

### 5.5 Ethical considerations and clinical integration

For clinical deployment, one can easily slot this framework into naming therapy routines. The system sorts each patient’s response and generates two A-CAM visualizations: one shows how the model made its prediction, and the other highlights patterns linked to aphasia. With these visual maps, clinicians get practical support; they can spot phonological therapy targets, monitor how production quality changes from session to session, and fine-tune intervention intensity. But this is not just plug-and-play; researchers still need to run prospective validation studies with real clinicians to check the system’s usability and clinical value.

Importantly, the framework is designed to enhance, not replace, clinical judgment. Key ethical considerations include: (i) the risk of automation bias, where clinicians may rely too heavily on A-CAM visualizations without independent verification; we emphasize these maps should be seen as indicative patterns, not diagnostic labels; (ii) training and evaluation solely on English-language AphasiaBank data, with cross-linguistic applicability not yet established; (iii) the need to adhere to data protection regulations given the sensitive nature of clinical recordings; and (iv) unanalyzed performance variation across demographic groups (age, gender, education), which presents an important equity issue for future research.

## 6 Conclusion

This paper addressed keyword-level recognition in aphasic speech by introducing a severity-aware classification model (AQFormer) and an aphasia-informed explanation approach (A-CAM). AQFormer conditions intermediate representations on Aphasia Quotient (AQ) severity via Feature-wise Linear Modulation (FiLM), supporting patient-sensitive modelling across impairment levels. A-CAM provides time–frequency attributions through complementary streams that separate evidence supporting the keyword decision from impairment-linked patterns, with the latter constrained by an aphasia filter incorporating pause, phoneme, and boundary-related cues. On an AphasiaBank-derived keyword dataset, the proposed framework improved recognition performance and calibration relative to severity-agnostic baselines and produced explanations that were more faithful, stable, and compact than standard Grad-CAM variants, with attribution concentrated in linguistically plausible regions. As corpus-derived annotations serve as a silver-standard reference, clinician-in-the-loop validation, evaluation on continuous speech, and cross-dataset robustness analyses form important directions for future work. Overall, the proposed framework demonstrates how severity-aware modelling and disorder-informed attribution can advance transparent, impairment-aware speech technologies for pathological speech with communication disorder research and practice.

## Data Availability

Publicly available datasets were analyzed in this study. This data can be found at: AphasiaBank.
