# Correction: AQFormer: severity-aware transformer with aphasia-specific CAM for spoken keyword classification in aphasic speech

**DOI:** 10.3389/frai.2026.1876574

**Published:** 2026-05-26

**Authors:** Gowri Prasood Usha, John Sahaya Rani Alex

**Affiliations:** School of Electronics Engineering, Vellore Institute of Technology, Chennai, Tamil Nadu, India

**Keywords:** aphasia, AphasiaBank, class activation mapping, explainable AI, film, keyword classification, severity-aware modeling, transformer

In the published article, there was a mistake in the **Funding** statement. The author(s) declared that financial support was not received for this work and/or its publication.

The correct statement reads: The author(s) declared that financial support was received for this work and/or its publication. The APC was paid by Vellore Institute of Technology, Chennai, India.

The original version of this article has been updated.

